# Parliament and the Profession

**Published:** 1917-04-07

**Authors:** 


### PARLIAMENT AND THE PROFESSION.

### Army Medical Service.

Colonel M'Calmont, in a question to the Under-Secretary of State for War, called attention to the case of a young civilian doctor, medically unfit for military service, who had been in medical charge of troops in his locality before the war and has now been replaced by another civilian doctor over military age. Mr. Macpherson explained that local service could not now be utilised; young medical men who are unfit are employed on general service at home, and must volunteer for service in any part of the Home Command.

### Army Dental Surgeons.

Mr. Macpherson informed Sir Charles Seely that the number of commissioned dental surgeons is 454.

### Motor Operating Theatres.

Sir W. Collins, inquiring of the Under-Secretary of State for War as to the use made of motor operating theatres, was informed by Mr. Macpherson that the more mobile character of the operations on the Western Front have been duly anticipated, and the full equipment for an operating theatre, together with the temporary theatre itself, can be sent to the Front with the *personnel* of a casualty clearing station, wherever it is required in any part of the line.

### Cerebral Meningitis at Reading.

Mr. Macpherson informed Captain Douglas Hall that there had been two fatal cases of cerebro-spinal meningitis during the recent outbreak at the jam factory, Coley, near Reading, then occupied by the School of Technical Training.

### Hospital Accommodation at Sandhurst.

Mr. Macpherson, in answer to a question by Mr. Evelyn Cecil, stated that extra hospital accommodation has been provided at Sandhurst, and during the recent outbreak of mumps certain of the halls of study were also utilised. There have been two deaths within the last four weeks—one from cerebro-spinal fever and the other from pneumonia.

### Venereal Diseases.

Mr. Hayes Fisher stated that 57 out of 145 local authorities in England and Wales which are responsible for the provision of free treatment for venereal diseases have not yet formally submitted schemes to the Local Government Board, but in all these cases schemes are under consideration.

